# New alternatives in photoprotection: Preparation and evaluation of lamellar silicate derivatives and their use as sunscreens

**DOI:** 10.1111/ics.70008

**Published:** 2025-09-10

**Authors:** Daniel Mabundu Kibwila, Alice Simon, Letícia Coli Louvisse de Abreu, Carlos Rangel Rodrigues, Valeria Pereira de Sousa, Marcelo de Pádula, Lucio Mendes Cabral

**Affiliations:** ^1^ Department of Pharmaceutics and Medicines, Faculty of Pharmacy Federal University of Rio de Janeiro Rio de Janeiro Brazil; ^2^ Laboratory of Instrumental Analysis, Duque de Caxias Campus Federal Institute of Rio de Janeiro Rio de Janeiro Brazil

**Keywords:** nanocomposite, new approach methodology, octyl methoxycinnamate (OMC), photoprotection, safety testing, Viscogel S4®

## Abstract

**Objective:**

The objective of this work is to investigate different sunscreens and Viscogel group organoclays for the preparation of new intercalated sunscreens to improve the effectiveness and safety in photoprotection using new approach methodology (NAMs).

**Methods:**

For this study, we examined Diethylamino hydroxybenzoyl hexyl benzoate (DHHB), octyl methoxycinnamate (OMC), Bemotrizinol (BEMT) and Viscogel S4®, S7®, and B8® using a set of *Saccharomyces cerevisiae* mutant strains that are sensitive to UVA, UVB and Solar Simulated Light (SSL) to evaluate their photoprotective and mutagenic potential. Additionally, we developed delaminated nanocomposites by chemical intercalation reactions followed by ultrasonic treatment to enhance clay exfoliation. These nanocomposites were then characterized using X‐ray Powder Diffraction (XRPD), Differential Scanning Calorimetry (DSC), Thermogravimetric Analysis (TGA) and Dynamic Light Scattering (DLS).

**Results:**

As a function of photoprotective action and safety, the combination of Viscogel S4® and OMC filter was chosen for nanocomposite development. Our results evidenced the intercalation of the OMC in the organoclay (S4 + OMC‐NC). Survival and mutagenesis tests indicated a positive effect of the formulation, as it increased cell survival to SSL, even without reducing mutagenesis, while its respective physical mixture was unable to provide antimutagenic protection.

**Conclusion:**

S4 + OMC‐NC showed promising results for photoprotection, offering cellular protection combined with antimutagenic effects upon SSL exposure. This formulation enables prospects for the preparation of new, effective and safe photoprotective formulations.

## Graphical abstract

The use of nanotechnology in sunscreens has increased, with various filters and organoclays tested for new intercalated formulations. As animal toxicity tests decrease, New Approach Methodologies are favoured. The S4 + OMC‐NC improved cell survival under SSL and showed antimutagenic properties, making it a promising candidate for safe photoprotection.

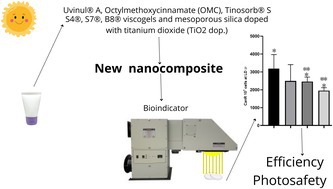

## Résumé

**Objectif:**

L'objectif de ce travail de recherche est d'étudier différents écrans solaires et organo‐argiles du groupe Viscogel pour la préparation de nouveaux écrans solaires intercalés, afin d'améliorer l'efficacité et la sécurité de la photoprotection à l'aide d'une nouvelle approche méthodologique (NAM).

**Méthodes:**

Pour cette étude, nous avons examiné le benzoate d'hexyle hydroxybenzoyle diéthylamino (DHHB), le méthoxycinnamate d'octyle (OMC), le bémotrizinol (BEMT), et le Viscogel S4®, S7® et B8® à l'aide d'un ensemble de souches mutantes *S. cerevisiae* sensibles aux UVA, UVB et à la lumière simulée (Solar Simulated Light, SSL), afin d'évaluer leur potentiel photoprotecteur et mutagène. De plus, nous avons développé des nanocomposites délaminés par des réactions d'intercalation chimique suivies d'un traitement par ultrasons pour améliorer l'exfoliation de l'argile. Ces nanocomposites ont ensuite été caractérisés à l'aide de la diffraction des rayons X sur poudre (X‐ray Powder Diffraction, XRPD), de la calorimétrie à balayage différentiel (Differential Scanning Calorimetry, DSC), de l'analyse thermogravimétrique (Thermogravimetric Analysis, TGA) et de la diffusion dynamique de la lumière (Dynamic Light Scattering, DLS).

**Résultats:**

En fonction de l'action photoprotectrice et de la sécurité, l'association du filtre Viscogel S4® et de l'OMC a été choisie pour le développement des nanocomposites. Nos résultats ont mis en évidence l'intercalation de l'OMC dans l'organo‐argile (S4 + OMC NC). Les tests de survie et de mutagenèse ont indiqué un effet positif de la formulation, car elle augmentait la survie cellulaire à la SSL, sans pour autant réduire la mutagenèse, tandis que son mélange physique respectif n'était pas en mesure de fournir une protection antimutagène.

**Conclusion:**

S4 + OMC NC a montré des résultats prometteurs pour la photoprotection, offrant une protection cellulaire associée à des effets antimutagènes lors de l'exposition à la SSL. Cette formulation offre des perspectives pour la préparation de nouvelles formulations photoprotectrices efficaces et sûres.

## INTRODUCTION

Ultraviolet radiations are generally related to harmful health effects such as direct damage to RNA and DNA, lipid oxidation and production of free radicals that can cumulatively generate burns, immunosuppression, photoaging and photocarcinogenesis [1]. These effects are due to the incident ultraviolet radiations on the Earth's surface. Among those, UVA radiation (315–400 nm) has the higher incidence, about 95%, followed by UVB radiation (280–315 nm) with 5% and without any terrestrial incidence of UVC (100–280 nm) because it is totally absorbed by molecular oxygen and by the atmosphere's ozone layer [2, 3]. Thus, the light emitted by a solar simulator is a source of radiation close to natural sunlight, but without the disadvantages linked to seasonal variations in irradiance throughout the year, offering UVA and UVB in proportions almost equivalent to natural terrestrial incidence.

Lesions caused by UVA and UVB radiation to DNA are varied, including the formation of photoproducts such as cyclobutane pyrimidine dimers (CPDs) and pyrimidine (6‐4) pyrimidone (6‐4PPs), oxidative lesions by base modifications such as 8‐oxoG, thymine glycol, 5,6‐dihydrothymine and the direct or indirect breaks of DNA strands. Faced with these DNA damages, the cell can trigger repair mechanisms such as base excision (BER), nucleotide excision (NER) or other mechanisms in order to circumvent the damage and survive, regaining all its initial functionalities. However, in the absence of repair or after an incorrect repair, the cell can undergo a mutation that can lead to a carcinogenic process or even, ultimately, cell death. As for DNA, RNA can suffer harmful actions of these UVA and UVB radiation, implying a change in genetic expressions that can cause the formation of proteins called non‐functional and promote cell senescence or the development of melanoma [4, 5, 6, 7].

Furthermore, these radiations are generators of reactive oxygen species (ROS) which are generally responsible for DNA single‐strand breaks. But if this single break is close to another ROS‐induced lesion, a double‐strand break can be produced. Therefore, photoprotection is necessary to prevent these damages, and there are many filters used for this purpose, as organic sunscreens that absorb UV radiation and inorganic sunscreens that reflect or scatter. Both are considered passive sunscreens. Recently, active action filters have been associated with them, which in addition to absorbing, reflecting or dissipating radiation, neutralize the free radicals generated by these radiations [8, 9].

The application of nanotechnology in photoprotection has been growing increasingly, in view of the great benefits offered, such as an increase in substantivity and surface area of contact by using clays that encapsulate some organic and/or inorganic filters, improving the sun protection factor of formulations, which can reduce the mutagenic risks related to the contact of these filters with radiation [10, 11]. This is the case of montmorillonite (MMT) clay or sodium (Na^+^) MMT, which has good ion exchange capacity and the possibility of reacting with organic compounds.

Organophilic derivatives of (Na^+^) MMT are usually obtained by intercalation reactions of quaternary ammonium salts into the lamellae. These organoclays, also known as Viscogel S4® (VS4), Viscogel S7® (VS7) and Viscogel B8® (VB8) were produced by replacing the interlayer Na^+^ with [bis(hydrogenated tallow alkyl) dimethyl ammonium], [dimethyl benzyl hydrogenated tallow ammonium] and [bis(hydrogenated tallow alkyl) dimethyl ammonium‐2‐propanol (10%)], respectively [12, 13].

Viscogel group organoclays have been studied for the possibility of incorporating or adding other organic components to their molecular structure, particularly organic sunscreens. Diethylamino hydroxybenzoyl hexyl benzoate (DHHB) (commercial name Uvinul® A), octyl methoxycinnamate (OMC) and Bemotrizinol (BEMT) (Commercial name Tinosorb® S) (Table 1) are organic filters representing each range of the ultraviolet region. While DHHB absorbs especially in the UVA region around 345 nm, OMC absorbs mostly in the UVB region with little UVA absorption, between 280 and 310 nm. BEMT, a synthetic molecule of high molecular weight, has a broad spectrum, absorbing between 290 and 400 nm, both in the UVA and UVB regions [14].

**TABLE 1:** Physical–chemical parameters of organic filters.

Name	DHHB	OMC	BEMT
Chemical structure	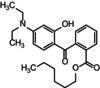	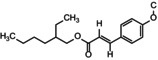	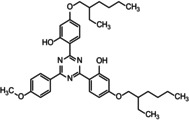
Molecular weight	397.515 g mol^−1^	290.397 g mol^−1^	627.826 g mol^−1^
Absorption spectrum	UVA	UVA + UVB	UVA + UVB

*Note*: See Reference [14].

The combination of organic and inorganic filters in a photoprotective formulation offers an increase in their photoprotector quality by protecting from both UV regions. Mesoporous silica doped with titanium dioxide (TiO_2_ dop.) presents itself as a good example, since it contains titanium dioxide, a micrometric inorganic filter widely known for its high photoprotective efficacy; it has a mesoporous structure capable of storing other actives as organic filters [15, 16].

Presently, the utilization of toxicity tests involving animals is diminishing, and there is an encouragement for the adoption of New Approach Methodologies (NAMs). These NAMs are defined as any technology, methodology, approach or combination thereof capable of imparting information pertaining to chemical risks while avoiding the necessity for animal experimentation. Techniques such as in silico, in vitro and ex vivo experiments may be employed within this purpose [17].

The yeast *Saccharomyces cerevisiae* is an eukaryotic and unicellular microorganism that has different strains used in biological studies to assess the toxicity, mutagenicity and genotoxicity caused by solar radiation. It was also used to evaluate the photoprotection of the components of a sunscreen. The mutant strain *Ogg1* is devoid of the OGG1 gene, responsible for DNA repair, through the mechanism based on base excision, in view of the 8‐OhdG (8‐hydroxy‐2′‐deoxyguanosine) lesions produced by ultraviolet radiation [9, 18, 19, 20, 21]. This strain is genetically modified and very biorelevant in photoprotection studies and in photomutagenic processes because it is sensitive to oxidative stresses [15, 16].

Thus, in this study, we aimed at the preparation and evaluation of new nanometric photoprotective actives obtained by DHHB, OMC and BEMT intercalation in pre‐formed micro/nanometric systems VS4, VS7, VB8 and TiO_2_ dop. Both efficacy (cell survival) and safety (mutagenesis) assessments were performed for these photoprotective compounds using the *Ogg1* mutant strain (CD138) of *S. cerevisiae* to confirm their potential use in photoprotection.

## MATERIALS AND METHODS

### Materials

VS4, VS7 and VB8 were purchased from Bentec (Livorno, Italy). Mesoporous silica doped with titanium dioxide was purchased from Sigma‐Aldrich (St. Louis, Missouri). DHHB, OMC and BEMT were purchased from Fagron (Rio de Janeiro, Brazil); Polysorbate 80 and Canavanin were purchased from Sigma‐Aldrich (Rio de Janeiro, Brazil).

### Photoprotective and antiphotomutagenic potential assessment using NAMs

#### Yeast strain *Ogg1* (CD138), media, growth conditions and cell treatments

The *Ogg1::TRP1* (CD138) mutant strain used in this work was derived from *S. cerevisiae* (wild‐type strain FF18733) is prototrophic for arginine. It was grown in 10.0 mL of yeast peptone dextrose (YPD) medium under agitation for 24 h at about 30°C. Then, 100.0 μL was spiked into another 10.0 mL of YPD, under stirring for 48 h at about 30°C, until the stationary phase. The culture was centrifuged for 5 min at 188 g, repeated 3 times and resuspended in 10 mL of sterile water. Subsequently, the optical density at 600 nm was verified. The cells were diluted to 10^7^ cells mL^−1^, which corresponds to an optical density equal to 1. Then, an aliquot of these cells, containing 100 μg mL^−1^ of each substance or mixture, was plated in a YPD solid medium in duplicate for evaluation of survival or in yeast nitrogen base‐dextrose (YNBD) solid medium with 60 mg L^−1^ canavanine for mutagenesis test for the selection of canavanine‐resistant (Can^R^) mutants after treatment. The treatment was in the dark to evaluate the cytotoxicity or mutagenicity of the substances without any irradiation and in the presence of solar simulator light (SSL) [15, 16].

In the dark, the flask containing a cell density of 10^7^ cells mL^−1^, was exposed to 100 μg mL^−1^ of each material in sterile water and was placed under agitation for 6 h at about 30°C; then, every 1 h, an aliquot was taken and plated.

#### Radiation source and dosimetry

Simulated solar light (SSL) irradiations were performed using a solar simulator (SS) (Oriel Model 91192‐1000, Newport Corp., USA) emitting 21.7 J m^−2^ s^−1^ UVA and 1.58 J m^−2^ s^−1^ UVB. Treatment was performed with increasing doses of SSL as previously described [16, 22, 23].

The dosimetry was measured using a VLX‐3‐W dosimeter (Vilber Lourmat, Marne‐la‐Vallée, France) with CX‐312 photocells. SSL doses (kJ m^−2^) and their respective irradiation times were: 0 kJ m^−2^ (0′00″), 2.5 kJ m^−2^ (34′42″), 3 kJ m^−2^ (41′39″), 4 kJ m^−2^ (55′35″), 5 kJ m^−2^ (1 h 9′25″), 7 kJ m^−2^ (1 h 44′9″) and 10 kJ m^−2^ (2 h 18′52″). After each dose, aliquots were taken, properly diluted in sterile water and plated on YPD medium in duplicate. After approximately a period of 4 days at 30°C, colonies were counted [15] and a graph was plotted. All experiments were performed in triplicate, expressed as averages with respective standard deviations. Mutagenesis in both treatments was evaluated only at the 37% lethal dose (DL_37_), a dose conventionally documented and based on statistical calculation, which guarantees the occurrence of an average lethal stroke per cell [15, 16, 24].

### Preparation of intercalated OMC‐nanocomposite and physical mixture

Nanocomposite (NC) was obtained by the solution intercalation method in a 2:1 ratio (OMC:clay), after optimizing the experimental conditions previously performed in our research group as previously described [12, 15]. First, the organophilic clay was dispersed in methanol under magnetic stirring for 30 min. Subsequently, the filter was solubilized in the same solvent and stirred until complete solubilization; then, this solution was added to the clay dispersion and stirred under reflux for 72 h at 60°C. Afterwards, the flask containing the mixture was stored at 0–8°C for 24 h. After this time, the mixture was centrifuged at 8073 g at 15°C for 1 h and the supernatant was collected in a 100.0 mL volumetric flask. The NC obtained was washed 3 times with 20 mL portions of methanol. Finally, the supernatants were collected and added to a 100 mL volumetric flask. Intercalation yields were indirectly calculated in the supernatant by UV–Vis spectrophotometry using a Perkin Elmer Lambda 35 spectrophotometer at 311 nm. Then, the precipitated material was lyophilized and stored for characterization tests. NC was obtained in triplicate.

The physical mixture (PM) was carried out following the same 2:1 ratio (filter: clay or TiO_2_ dop.) using a glass plate and adding drops of methanol. After mixing, the PM was dried at 50°C for approximately 2 h. The PM was stored for characterization tests.

### Characterization of intercalated OMC‐nanocomposite and physical mixture

Raw material and their respective PM were used as negative controls for evaluating the formation of NCs. Powder X‐ray diffraction (PXRD) was performed using the Shimadzu XRD 6100 diffractometer (Tokyo, Japan) operated at 40 kV and 30 mA. The diffraction angle 2θ was used between 2 and 60° at room temperature using CuKα wavelength (1.542ª) as the X‐ray source. Bragg's equation was used to measure basal spacing. Fourier‐transform infrared spectroscopy (FTIR) analysis was carried out using the Shimadzu IR‐21 Prestige spectrophotometer (Tokyo, Japan) and samples (1.0% w/w) were prepared in KBr disk. Thermogravimetric analysis was performed on a Shimadzu TGA‐60 Thermal Analyzer (Tokyo, Japan) to evaluate mass loss in an aluminium pan with a nitrogen flow rate of 50 mL min^−1^, temperature range between 30 and 500°C and temperature rate maintained at 10°C min^−1^. Differential scanning calorimetric (DSC) analysis was carried out using the Shimadzu DSC‐60 thermal analyzer (Tokyo, Japan). Samples were placed into 40 mL sealed aluminium pans and scanned over a temperature range of 25 and 350°C with a scanning rate of 10°C min^−1^ under a nitrogen flow rate of 50 mL min^−1^. The particle size was determined by Dynamic Light Scattering (DLS). The size distribution of the new nanocomposites was measured with a Malvern Mastersizer 2000/2000E particle size analyser. The samples (VS4, VS7, VB8 and TiO_2_ dop.) were previously dispersed in 20 mL of an aqueous solution with 0.1% (w/v) 80 and then taken to ultrasound for 10 min to disperse the particles. The analysis was performed in triplicate and expressed as averages with respective standard deviation.

### Statistical analysis

Statistical analysis of the results was performed using the GraphPad Prism software (version 8.0.2), in at least triplicate independent experiments. For data with non‐parametric distribution, as in survival analysis and direct mutagenesis – Can^R^, ANOVA tests were performed, followed by the Kruskal–Wallis test, comparing the averages and standard deviations of the results and adopting a significant difference when *p* < 0.05.

## RESULTS AND DISCUSSION

### Cyto and mutagenic profile of lamellar structures and filters as supplied

The insertion of sunscreens in nanometre‐sized systems provides several benefits in derivative formulations, not only increasing the stability of the active, but also increasing the formulations substantivity and as well as greater efficiency in the absorption process of light fundamental to the effectiveness of a sunscreen [25, 26]. In addition, the nanometric size promotes an increase in the surface contact area, allowing a large number of particles per unit weight to be obtained at the application site, which improves the physicochemical properties of these nanostructures [27, 28].

However, with the expanded commercial offer of sunscreen formulations and considering that these sunscreen filters must remain on the skin and not penetrate the systemic circulation, there is a concern about the toxic potential related to transdermic absorption of these substances and the generation of free radicals that may be harmful. The intercalation of the photoprotective agent in organophilic clays, as observed in this work, not only prevents the formation of free radicals by stabilizing these substances but also prevents transdermal absorption due to the external hydrophilic clay surface [29, 30].

**FIGURE 1:**
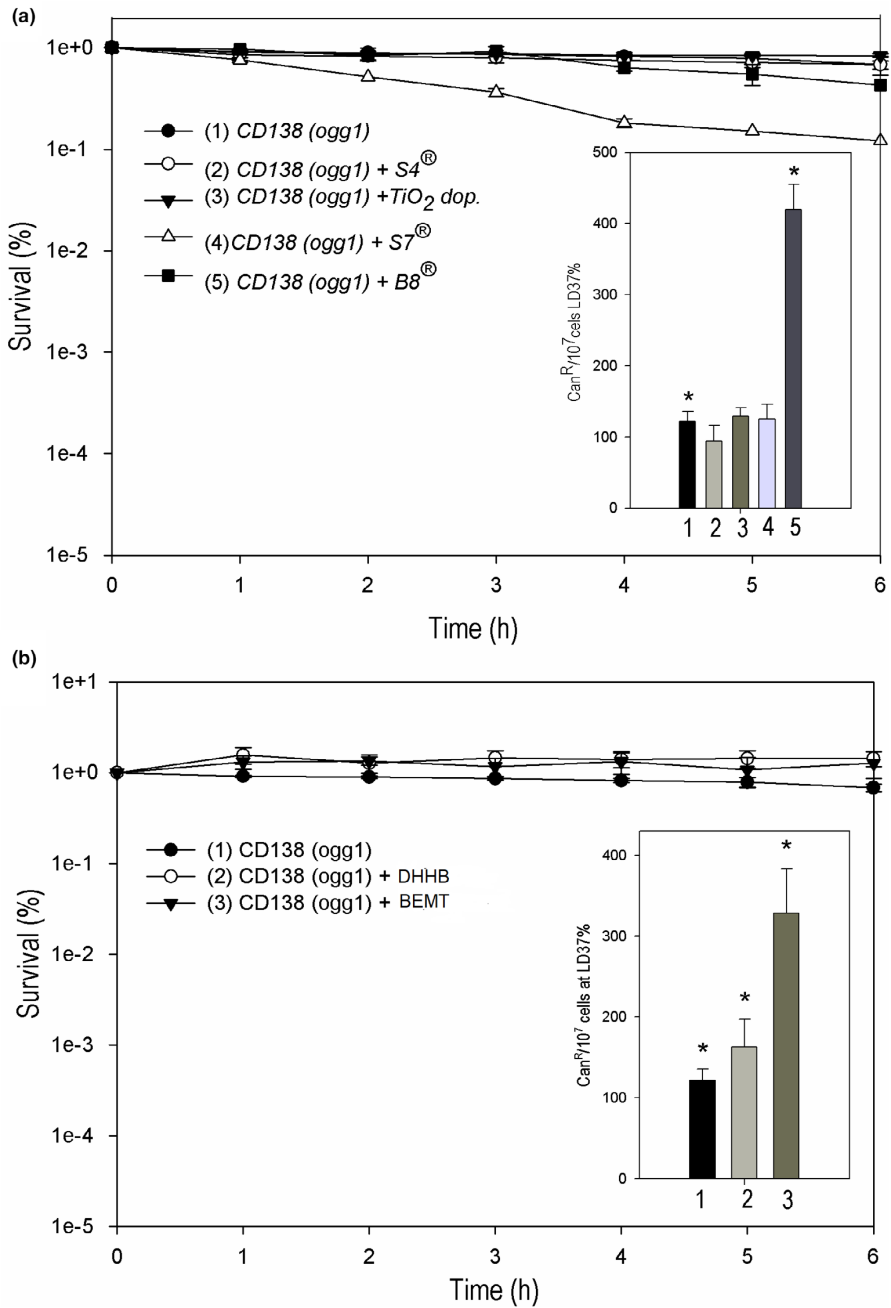
(a) Survival (%) and spontaneous mutagenesis (Can^R^/10^7^ cells at LD_37%_) of CD138 mutant strain (*Ogg1*) in the dark to assess the cytotoxicity of Viscogels S4®, S7®, B8® and TiO_2_ dop. (100 μg mL^−1^) during 6 h of treatment. Data represent means ± SE. *Different (*p* < 0.05) from control (CD138 strain) and among each other. (b) Survival (%) and spontaneous mutagenesis (Can^R^/10^7^ cells at LD_37%_) of CD138 mutant strain (*Ogg1*) in the dark to assess the cytotoxicity of organic sunscreen molecules—DHHB and BEMT (100 μg mL^−1^) during 6 h of treatment. Data represent means ± SE. *Different (*p* < 0.05) from control (CD138 strain) and among each other.

The phototoxicological evaluation of these new nanocomposites is essential to ensure their effectiveness and safety. In this work, these new photoprotective nanocomposites were prepared exploring the reactivity of the lamellae of different types of silicates as Viscogel derivatives and the pores of mesoporous silica. Each pre‐formed silicate‐lamellar structure, before intercalation with chemical sunscreen, was isolated and tested for cytotoxic and mutagenic (Figure 1a) profiles in the dark on the *Ogg1* strain. The strain was not sensitive to VS4, VB8 and TiO_2_ dop. samples. However, the VS7 sample was toxic in yeast since there was a decay of the survival fraction (*p* < 0.05). This cytotoxicity of VS7 can be explained by the presence of the aromatic ring in the chemical structure of the alkylammonium of this organoclay, capable of accelerating the cell death mechanism of the *Ogg1* strain [31].

Yeast, being an eukaryotic model and equipped with genetic material orthologous to humans, is useful as a robust and efficient in vitro screening test to reveal spontaneous toxic or non‐toxic reactions of the actives of a formulation [15, 32, 33, 34]. Considering mutagenicity without any irradiation, VS7 and TiO_2_ dop. were not mutagenic as they were statistically equal in mutation frequencies of VB8, on the contrary, showed an increase in the frequency of mutants (*p* < 0.05), indicating a mutagenic effect in the dark. Therefore, both VS7 and VB8 were discarded as candidates for intercalation of organic sunscreens due tocytotoxic and mutagenic effects, respectively. In contrast, VS4 was antimutagenic (*p* < 0.05) compared to the control (CD138) when evaluating the generated mutant frequency. In parallel, the organic filters elected for the intercalation process in the lamellar silicate, showed no applicable toxic effect against yeast, but in the same dark condition, only BEMT was mutagenic (*p* < 0.05) (Figure 1b). Cito and mutagenicity of the OMC had already been demonstrated in previous works, for that reason the OMC was not tested in this initial stage [35].

### Cyto and mutagenic profile of lamellar structures and filters after SSL irradiation treatment

**FIGURE 2:**
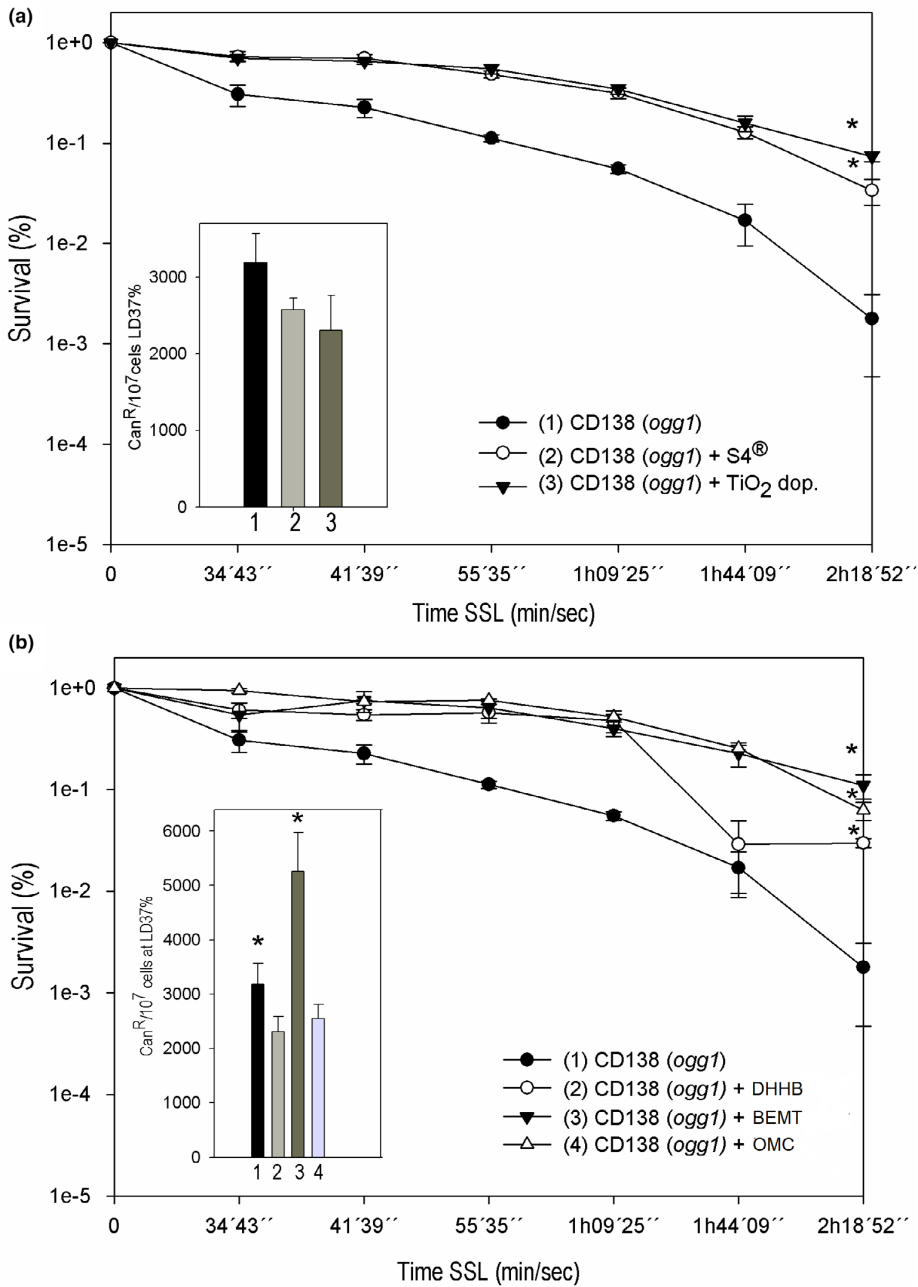
(a) Survival (%) and induced mutagenesis by SSL irradiations (Can^R^/10^7^ cells at LD_37%_) of the CD138 mutant strain (*Ogg1*) to assess the cytotoxicity of VS4® and TiO_2_ dop. (100 μg mL^−1^). Data represent means ± SE. *Different (*p* < 0.05) from control (CD138 strain) and among each other. (b) Survival (%) and induced mutagenesis by SSL irradiations (Can^R^/10^7^ cells at LD_37%_) of the CD138 mutant strain (*Ogg1*) to assess the cytotoxicity of organic sunscreen molecules—DHHB, OMC, BEMT (100 μg mL^−1^). Data represent means ± SE. *Different (*p* < 0.05) from control (CD138 strain) and among each other.

In the presence of SSL irradiations, the selected lamellar structures (VS4 and TiO_2_ dop.) were photoprotective, increasing the cell survival fraction (*p* < 0.05) (Figure 2a). This photoprotective action is due to the physical barrier against UV radiation offered by the lamellar or tubular packaging of these structures [36].

Increased survival was also seen with the use of organic filters in this study (DHHB, OMC and BEMT). They protected the yeast cells against the toxic effects of radiation emitted by the solar simulator (Figure 2b). The photoprotective action of filters is due to their mechanisms of radiation absorption and releasing them in the form of heat, with DHHB in the UVA range, OMC in the UVB range with a small absorption in the UVA range, and BEMT, a large molecular size filter that absorbs both, in the UVA and UVB ranges [14]. In addition, DHHB demonstrated its photodegradation around 2 h after exposure, thus reaffirming the need for mixing filters and reapplication of photoprotective formulations to increase their efficacy.

As for the mutagenic effects, the selected lamellar silicate structures (VS4 and TiO_2_ dop.) presented a level of induced mutagenesis equivalent to the control (*p* > 0.05) (Figure 2a), that is, in terms of photogenotoxicity, these structures were not mutagenic and not antimutagenic. This result was also seen with the use of DHHB and OMC filters. However, contrary to the other filters under study, BEMT proved to be photomutagenic as it significantly increased the frequency of mutants (*p* < 0.05) (Figure 2b), thus discarding its possibility of being intercalated in VS4. This increased mutation frequency can be explained by the high number of reactive points in the chemical structure of this BEMT filter, capable of generating several oxidative intermediates responsible for increasing mutagenesis [37]. BEMT has toxicophoric groups in its structure that can lead to the formation of intermediate reactive species and photomutagenic decomposition products such as singlet oxygen or free radicals.

In this way, due to the photoprotective action and safety evidenced by the lamellar structure of VS4 and by OMC chemical sunscreen, in which it was extensively studied in relation to its safety [38], the VS4 and OMC were chosen for nanocomposite preparation, even with a small contribution in the UVA range for OMC.

### Characterization of the S4/OMC‐nanocomposite and its respective physical mixture

The supernatant liquid of the intercalation process final product was used to indirectly measure intercalation yield by UV–Vis spectrophotometry. Starting from an initial mass of around 0.8 g of OMC per 0.4 g of VS4, the supernatant, after dilution, revealed a yield of around 34.23%. This yield was better than those obtained by Shen et al. [39], whose intercalated polyethylene oxide (PEO) in MMT Na + had a yield of 28%.

Organoclays, PM and NC revealed a monomodal distribution on a micrometric scale (Appendix S1). These sizes are the result of the agglomeration of different lamellar units, which generate, after the physical delamination process or promoted by the action of a solvent, nanometric‐sized systems. However, Viscogel derivatives disaggregate into nanometric structures in different organic solvents and formulations in which they can be incorporated. The solvent used in these materials for this analysis was distilled water with the addition of polysorbate 80 at 0.1% w/v, the main reason for not revealing their nanometric scale [40, 41]. However, the TiO_2_ dop. particles, which initially showed micrometric dimensions in an aqueous medium, did not demonstrate the possibility of regenerating a nanometric system when solubilized in toluene and isopropyl myristate, being excluded as an option for obtaining nanocomposites intercalated with chemical sunscreens.

The PM were prepared so as not to allow sufficient contact time between the reagents to observe the intercalation. Paiva et al. [15] had already described the insertion of chemical sunscreens in sodium montmorillonite when the reaction yields were compatible with those observed in this work. However, this approach was restricted to ionizable chemical filters soluble in acidic aqueous solutions. The evidence of obtaining the new systems was confirmed by the characterization techniques that are presented below.

**FIGURE 3:**
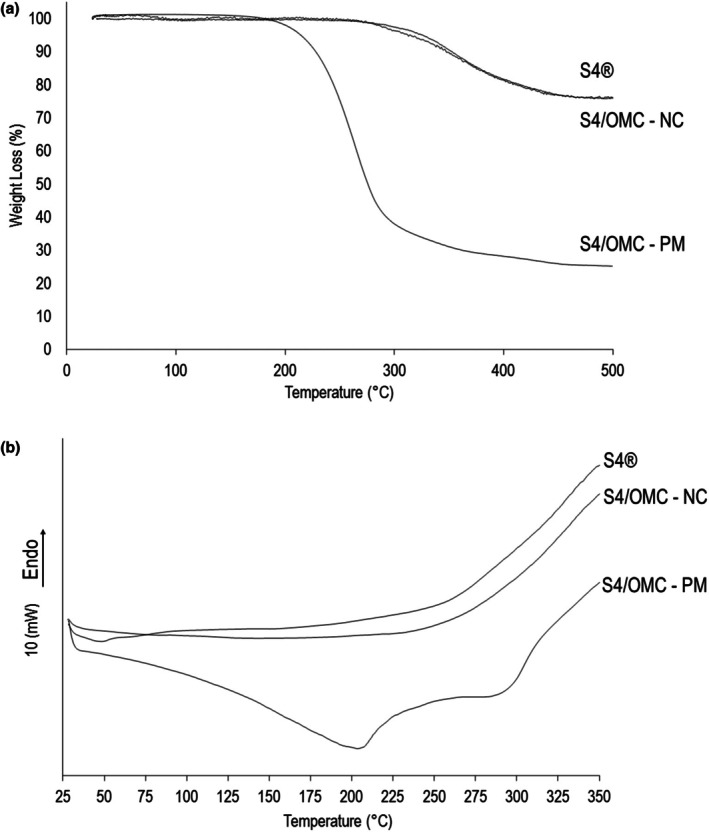
Thermal analysis: (a) TGA curves of VS4 as supplied (S4®), S4/OMC‐NC and S4/OMC‐PM; (b) DSC curves of VS4 as supplied (S4®), S4/OMC‐NC and S4/OMC‐PM.

In comparative terms, the TGA results of VS4 as supplied and the intercalation product showed no change in the stability of the samples since the loss of mass occurred in a single time, around the same range of temperature and followed by a prolonged stability phase that was equivalent (Figure 3a). The observed mass loss is related to the alkylammonium ion of the pure material and to the OMC organic filter, because the lost mass percentage is a consequence of the yield obtained in the intercalation process and alkylammonium content. As for the physical mixtures, the TGA analysis showed a considerable mass loss of the samples, being 72.319% only related to the alkylammonium ion of the VS4 [42]. To complete this information obtained with TGA, DSC analysis showed (Figure 3b) an endothermic event in VS4 as supplied, with initial temperatures up to 100°C, which practically do not appear in the intercalation product. This is due to the removal of water particles from the interlamellar spaces that are found in large amounts in VS4 as supplied. This initial data may indicate the formation of a new product after the intercalation process because the intercalated material reduces the passivity of water accommodation in the interlamellar space [43]. As in the analysis of the intercalation product, these TGA analyses of the mixtures were complemented by the appearances of endothermic events in the DSC analyses, confirming the achievement of the new systems. TGA and DSC techniques showed significant differences between the nanocomposite and the physical mixture. Due to the pasty appearance of the physical mixture formed, it was not used in the analysis of XRPD and FTIR.

**FIGURE 4:**
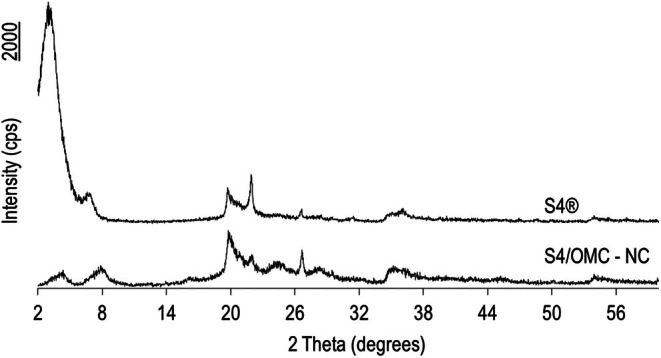
Diffractograms of VS4 as supplied (S4®) and S4/OMC‐NC nanocomposite.

In a different way, XRPD analysis demonstrated the appearance of one more peak in the intercalated sample, with a left shift, representing the presence of OMC in the interlamellar space that is not present in the VS4 as supplied (Figure 4). In this peak, located at 2θ = 4.43°, the interlamellar spacing calculated, after applying Bragg's law, is higher (*d* = 20.35 A°) than the VS4 as supplied (*d* = 11.63 A°) in its highest peak (2θ = 7.6°); that is, OMC inserted itself inside the interlamellar space, originating an intercalated nanocomposite, possibly oriented in the form of a monolayer.

**FIGURE 5:**
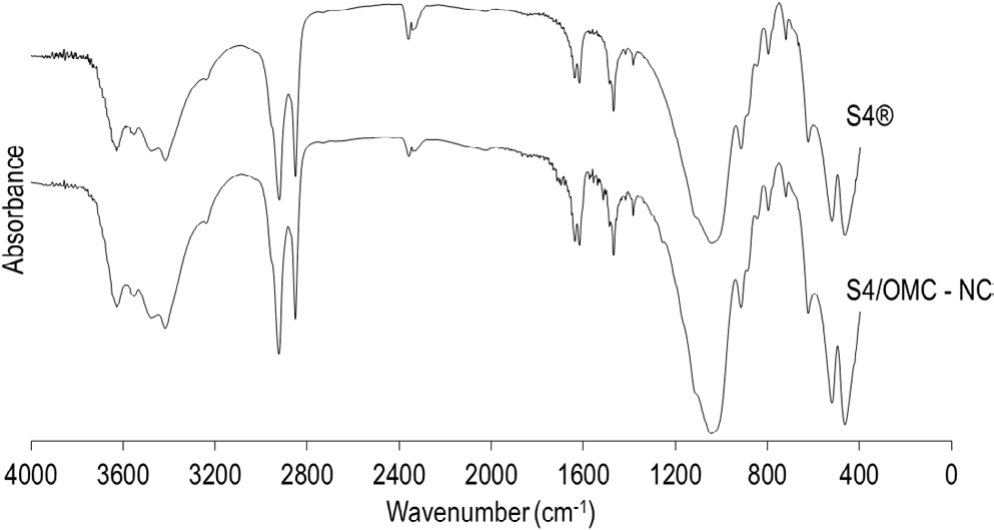
FTIR spectra of VS4 as supplied (S4®) and S4/OMC‐NC nanocomposite.

FTIR analysis did not clearly show a significant difference between the VS4 as supplied and the intercalation product (Figure 5) since the bands of the VS4 are larger than those of the OMC. So, this technique is not effective in detecting the newly formed system. However, there were slight differences around 1750 and 1463 cm^−1^ of the intercalation product, which could correspond respectively to the stretching of the C=O group of the OMC and the C=C group of its aromatic ring, as recorded by Daneluti et al. and Wu et al., thus identifying the presence of this filter in the intercalation product [37, 44].

### Cytotoxic and mutagenic profile of the S4/OMC‐nanocomposite and its respective physical mixture

**FIGURE 6:**
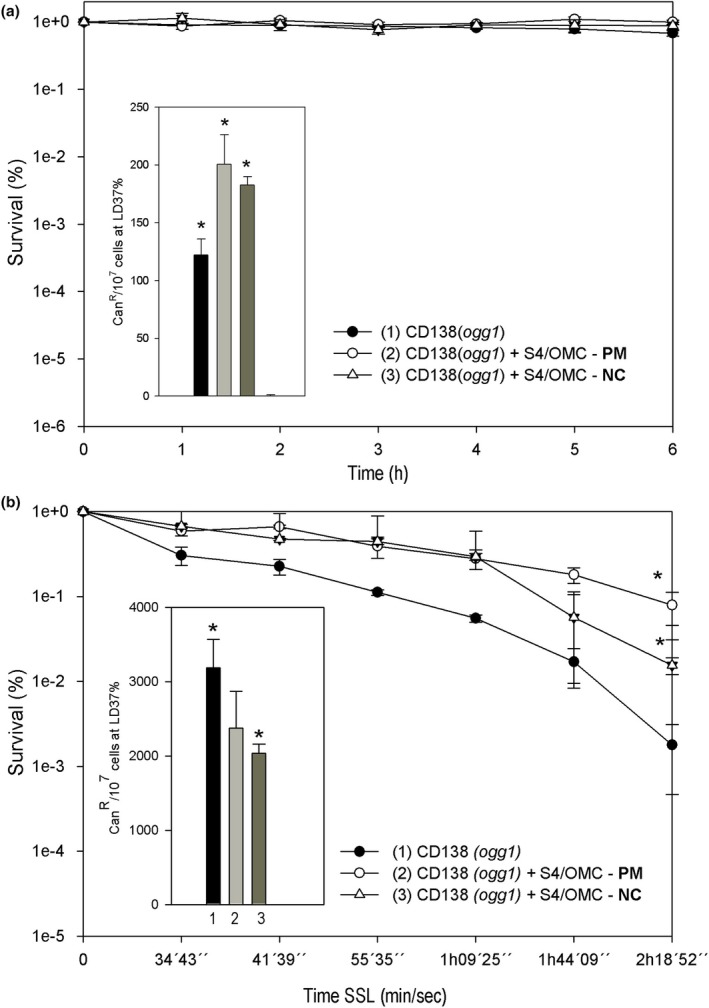
(a) Survival (%) and spontaneous mutagenesis (Can^R^/10^7^ cells at LD_37%_) of the CD138 mutant strain (*Ogg1*) in the dark to assess the cytotoxicity of the association of Viscogel S4® and OMC in the physical mixture (PM) and in the nanocomposite (NC) (100 μg mL^−1^). Data represent means ± SE. *Different (*p* < 0.05) from control (CD138 strain) and among each other. (b) Survival (%) and induced mutagenesis by SSL irradiations (Can^R^/10^7^ cells at LD_37%_) of the CD138 mutant strain (*Ogg1*) to assess the cytotoxicity of the association of VS4 and OMC in the physical mixture (PM) and in the nanocomposite (NC) (100 μg mL^−1^). Data represent means ± SE. *Different (*p* < 0.05) from control (CD138 strain) and among each other.

Yeast cells were challenged with intercalation product and/or with the prepared physical mixtures to determine cell survival and mutagenesis (Figure 6a). These results were considered interesting at this first moment because even with mutagenic effects in the dark, all samples proved to be non‐toxic to our cell model, thus requiring an investigation of these behaviours under simulated sunlight (SSL). Interestingly, a photoprotective effect on the yeast strain was observed when the nanocomposite and/or physical mixtures samples were used in front of SSL (Figure 6b). That is, they increased cell protection against the effects of UVA‐UVB radiation at all treatment points when compared to the control (only the strain without any sample (*p* < 0.05)). This photoprotection is due to the presence of photoprotective actives in these samples, mainly OMC that absorb or reflect or disperse these radiations. In addition, VS4 contributes to this photoprotection by offering a simply physical barrier against these radiations, allowing for greater reflection or dispersion given their high contact surfaces and large number of particles per unit of weight [45].

The set of NC and/or PM protected cells against the cytotoxic effects of CPDs and 6‐4PPs photoproducts induced by ultraviolet radiation coming from the SSL [46]. Cell survival began to decline in all samples around 1 h after exposure to SSL, probably because cell attack started not only by the radiation itself, but also by the products generated by these radiations such as reactive oxygen species (ROS), in addition to excess DNA repair and strand breaks [47, 48].

There are scientific studies that demonstrate that UV radiation emitted by a solar simulator in contact with nano‐sized actives such as OMC generates a series of reactive oxygen species (ROS) such as superoxide anion radical ($O_{2}^{\cdot -}$), hydrogen peroxide (H_2_O_2_), singlet oxygen (1O_2_) and ^−^OH via Fenton reaction or similar reactions, with an important role in DNA damage and leading to cytotoxicity [49, 50]. Even with a repair mechanism functioning in the face of oxidative stress, cells with high levels of DNA damage can saturate. These DNA damages can lead to homologous recombination that, during the process, can generate single strand breaks in the DNA, which, in a situation of repair saturation, can end up in double breaks introduced by the simultaneous repair action of the *Ogg1* enzyme. This justifies the cell lethality often observed, as in our case, in doses above 1 h. However, at lower doses of SSL, the *Ogg1* enzyme may be necessary for cell survival, avoiding the appearance of strand breaks [16].

The physical mixtures showed better photoprotective performance in the last points of treatments compared to the nanocomposite. However, this mixture, curiously, was neither more effective nor cytotoxic. In general, the reflection or absorption of UV radiation by the particles of the mixtures and/or the nanocomposite was more important than absorption and the consequent generation of ROS or other attacks on cells.

In photomutagenic terms, it was observed that none of the samples was photomutagenic; that is, all of them led to a reduction in the mutation frequency (Figure 6b). This reduction was significant with nanocomposite compared to the control (*p* < 0.05), which gives these associations an antiphotomutagenic character. Although the filter present in these samples can act as chromophores against UV radiation, increasing the formation of ROS [51, 52], their presence in organoclay structures S4 proved to be useful in attenuating the mutagenic effects of these filters in the presence of SSL.

When analysed with each other, the NC showed a greater antiphotomutagenic effect than the PM, as there was a statistically significant difference between the two samples. These results demonstrate the photoprotective and antiphotomutagenic effect of these samples and corroborate that demonstrated in the scientific study reported by Paiva et al. when they analysed TiO_2_ and octyldimethyl‐PABA (ODP) in association with clay [15]. These results reinforce what has already been said before about a good photoprotection of a formulation; it is necessary to guarantee efficiency together with safety, which is often obtained by the combinations of inorganic and organic filters, in addition to the association of other components with photoprotective activities such as organoclays and antioxidant agents.

In this sense, the materials tested in this work involved organic filter as OMC and layered silicate structures, also recognized for photoprotective activities. Herein, the nanocomposite obtained from OMC intercalation in organoclay VS4 was more effective both in photoprotection, offering the photoprotective efficacy of the cell and in mutagenicity, exhibiting a more important antiphotomutagenic activity compared to the other combinations.

## CONCLUSION

In this work, our results showed, among the studied lamellar structures, that VS4 is a promising candidate for photoprotection, since the increased cell survival upon SSL, even without reducing mutagenesis. However, after intercalation of OMC into VS4 and its respective physical mixture, the S4/OMC‐NC showed to be more promising than VS4 as supplied, in terms of efficacy (increased survival) and safety (decrease the frequency of mutagenesis). Thus, the S4/OMC‐NC was identified as the best candidate for photoprotection, as it ensured cellular protection combined with antimutagenic effects upon SSL, enabling prospects for the preparation of new effective and safe photoprotective formulations.

## CONFLICT OF INTEREST STATEMENT

None of the authors have a conflict of interest to disclose.

## Supporting information


Appendix S1:

## Data Availability

The data that support the findings of this study are available from the corresponding author upon reasonable request.
