# Supplementary material for: New alternatives in photoprotection: Preparation and evaluation of lamellar silicate derivatives and their use as sunscreens
Source: Int J Cosmet Sci. 2025 Sep 10;48(1):172–85. doi: 10.1111/ics.70008 (PMC12877990; doi:10.1111/ics.70008)
Supplement: Supplementary file 1 — Appendix S1: [file ICS-48-172-s001.docx]

Supplementary material.

Table 1. Particle size VS4

|  | Particle size (µm) | |  |  |
| --- | --- | --- | --- | --- |
| Samples |  |  |  | Span |
|  | d10 | d50 | d90 |  |
| 1 | 3,972 | 17,290 | 50,812 | 2,709 |
| 2 | 4,054 | 18,786 | 58,129 | 2,878 |
| 3 | 4,300 | 20,627 | 55,882 | 2,501 |

**Figure 1. Particle size distribution of VS4**

**
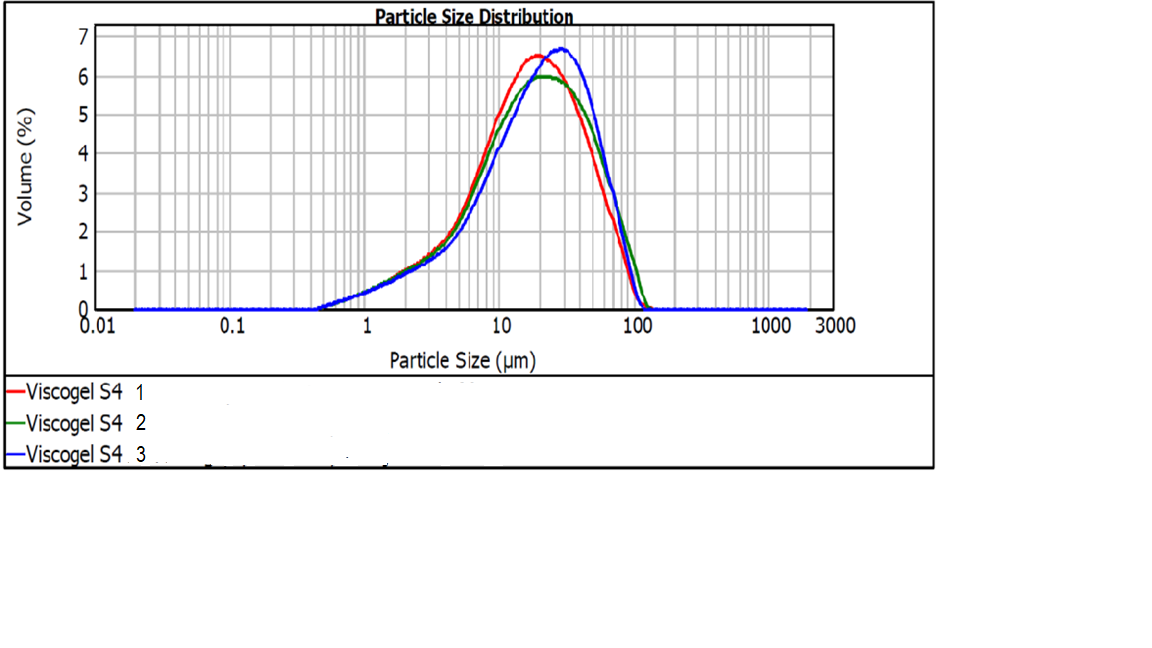
**

Table 2. Particle size VS7

|  | Particle size (µm) | |  |  |
| --- | --- | --- | --- | --- |
| Samples |  |  |  | Span |
|  | d10 | d50 | d90 |  |
| 1 | 7,633 | 44,995 | 100,544 | 2,065 |
| 2 | 5,371 | 32,596 | 81,066 | 2,322 |
| 3 | 6,096 | 35,786 | 87,386 | 2,272 |

**Figure 2. Particle size distribution of VS7**

**
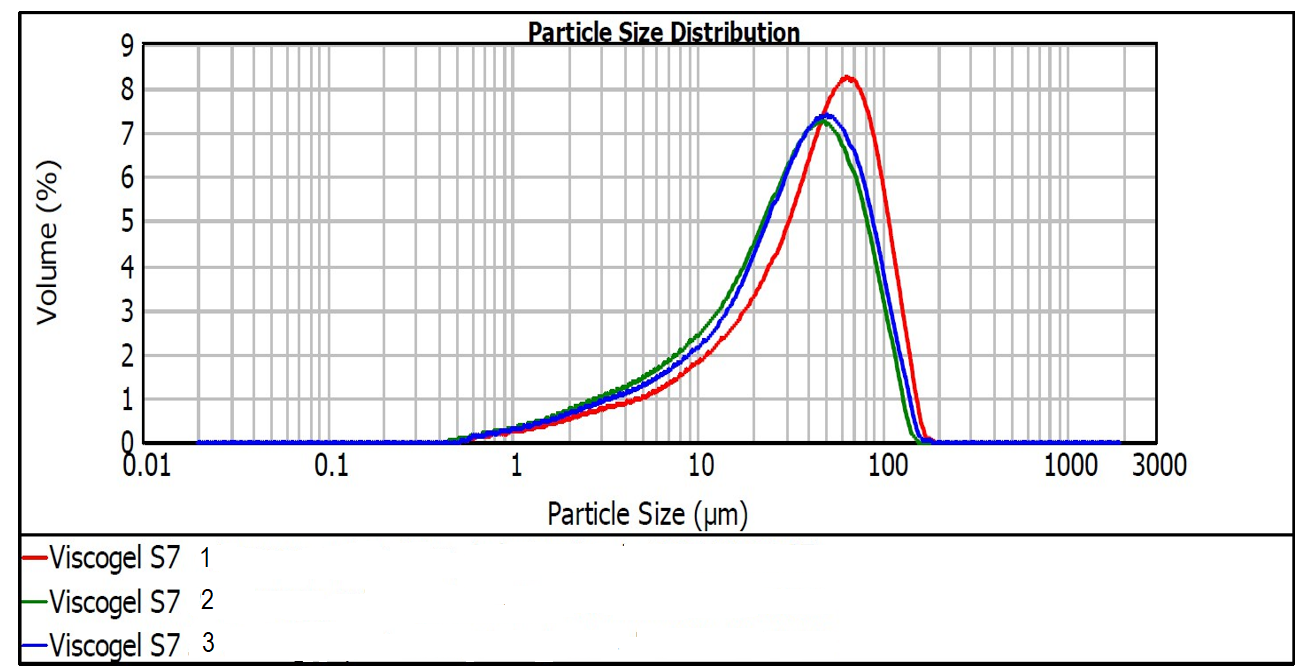
**

Table 3. Particle size VB8

|  | Particle size (µm) | |  |  |
| --- | --- | --- | --- | --- |
| Samples |  |  |  | Span |
|  | d10 | d50 | d90 |  |
| 1 | 3,227 | 22,400 | 52,680 | 2,208 |
| 2 | 4,395 | 27,434 | 64,010 | 2,173 |
| 3 | 4,396 | 23,618 | 56,884 | 2,222 |

**Figure 3. Particle size distribution of VB8**

**
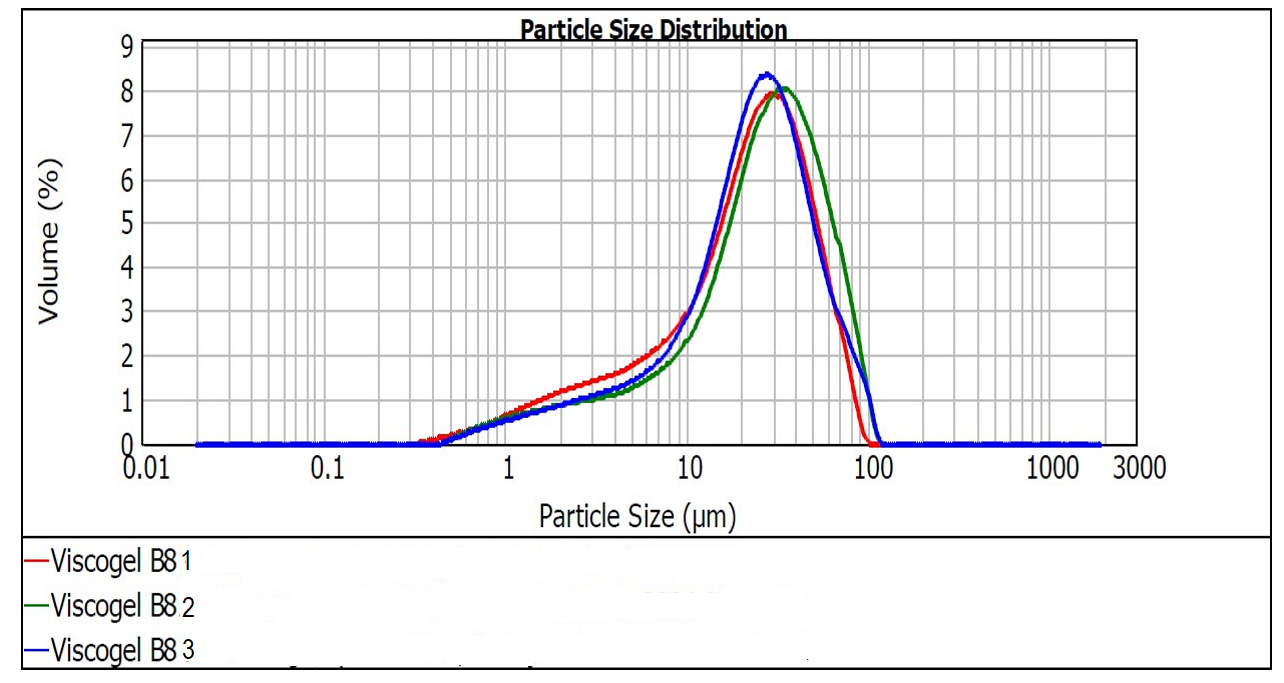
**
